# Interleukin-27 tackles immunosuppression in chronic lymphocytic leukemia

**DOI:** 10.1080/2162402X.2023.2276490

**Published:** 2023-11-03

**Authors:** Iria Fernandez Botana, Giulia Pagano, Etienne Moussay, Jerome Paggetti

**Affiliations:** a Tumor Stroma Interactions, Department of Cancer Research, Luxembourg Institute of Health, Luxembourg; b Faculty of Science, Technology and Medicine, University of Luxembourg, Esch-sur-Alzette, Luxembourg

**Keywords:** Cytokine, IL-27, CD8+ T cells, immunotherapy, leukemia

## Abstract

Chronic lymphocytic leukemia (CLL) is the most common adult leukemia in the western world. It is characterized by a high dependency on interactions with the surrounding immune landscape, highlighting its suitability for immune-mediated therapeutic interventions. We recently revealed that the cytokine IL-27 exerts a strong anti-tumor role in CLL through a T-cell-mediated mechanism.

## Main text

Harnessing the power of the immune system against cancer probably represents one of the most clinically meaningful scientific breakthroughs of this century in the field of cancer research. For solid and hematological malignancies alike, the immune landscape surrounding the tumor has emerged as a powerful modulator of malignant fate and patient prognosis. Consequently, it comes as no surprise that cytokines, the so-called messengers of the immune system, have received increasing research attention during the last decade, already yielding remarkable results in the clinic.^1^ Interleukin 27 (IL-27), a heterodimeric cytokine belonging to the IL-12 family, has been the subject of a number of recent studies investigating its involvement in cancer development and progression, generating truly significant but rather pleotropic results for distinct malignancies. Analogous to most immune mediators, whether IL-27 promotes or hinders malignant growth seems to be a matter of context.^2^ Nevertheless, given its clear effects on tumor progression, exploring the role of this cytokine in the setting of different cancer types represents an attractive research pursuit.

While most cancers interact with their surrounding environment to some extent, some malignancies heavily rely on these interactions to receive the signals necessary to survive and thrive. This is the case of chronic lymphocytic leukemia (CLL), an incurable hematological malignancy commonly regarded as the most prevalent adult leukemia in the western world. One of the defining features of CLL is its inability to survive *ex vivo* without mimicking the stimulation from its native microenvironment, highlighting the intrinsic importance of these complex interactions in the pathobiology of CLL.^3^ We previously performed the first characterization of the CLL tumor microenvironment using mass cytometry in the widely used Eµ-TCL1 mouse model of CLL, where we reported a deeply immunosuppressive environment at the tumor niche.^4^ Moreover, we evidenced the clinical potential of restoring effective tumor immunity in this malignancy by successfully reducing tumor load while targeting the immune checkpoints PD-1 and LAG3.^5^ Recently, we also characterized the T cell landscape within different immune compartments of CLL patients using multi-omics technology, and further explored the translational potential of immunotherapies for the treatment of this malignancy.^6^

Despite the need for the development of effective immunotherapies for the treatment of CLL, as well the recently unraveled promise of IL-27 in cancer therapeutics, the role of IL-27 in the development and progression of CLL remained largely unexplored. In our recent publication, we employed a range of transgenic mouse models as well as clinical samples to elucidate the role of this cytokine in the complex immune landscape in CLL.

Levels of soluble mediators are known to be disturbed in oncology patients depending on whether they favor or hinder tumor growth, increasing or decreasing respectively when compared to healthy individuals.^7^ Interestingly, our data^8^ uncovered a significant and consistent decrease in IL-27 serum levels as the disease progresses in both pre-clinical and patient CLL samples, suggesting an anti-tumor effect of IL-27 in the development and progression of this malignancy. To evaluate the translational implications of this observation, we conducted a series of pre-clinical trials by adoptively transferring TCL1 leukemic cells into C57BL/6 and transgenic mouse models, as well as through the neutralization of this cytokine *in vivo*. For this purpose, we employed a constitutive KO murine model of the IL-27 subunit EBI3 (also known as the EBI3KO mouse model), hence rendering cells unable to produce this cytokine. The EBI3KO model was used to generate its leukemic counterpart, the TCL1-EBI3KO model, which spontaneously develops CLL and lacks the production of IL-27. As expected, all the aforementioned *in vivo* studies reported an increased tumor growth in the absence of this cytokine, conclusively pointing toward a strong anti-tumor role of IL27 in CLL. The decrease in tumor load was accompanied by the partial restauration of the anti-tumor immune response, evidenced by a less immunosuppressive leukemic immune landscape in the presence of IL-27, and in consonance with our previous publications.

While previous investigations have reported a direct cytotoxic effect of IL-27 in several malignancies,^9^ our data strongly suggests that T cells are the main mediators in the observed IL-27-driven anti-tumor immunity in CLL. This is evidenced by the ability of isolated T cells to control CLL progression more efficiently in the presence of this cytokine in an immunodeficient mouse model. Of particular importance, patient-derived T cells treated with IL-27 displayed a higher cytotoxic ability toward leukemic cells than their untreated counterparts; highlighting the potential of this cytokine to reactivate anti-tumor immunity in CLL patients. Finally, gene expression profiling analysis of T cells in the presence and absence of IL-27 shed light on the molecular mechanism involved in this process, underlining the role of the synapse-formation-modulator profilin 1,^10^ and the amino acid transporter SLC7A5 among others. Identifying and connecting the molecular underpinnings of this pathway remains an exciting area of research that must be explored by future investigations.

**Figure 1.**
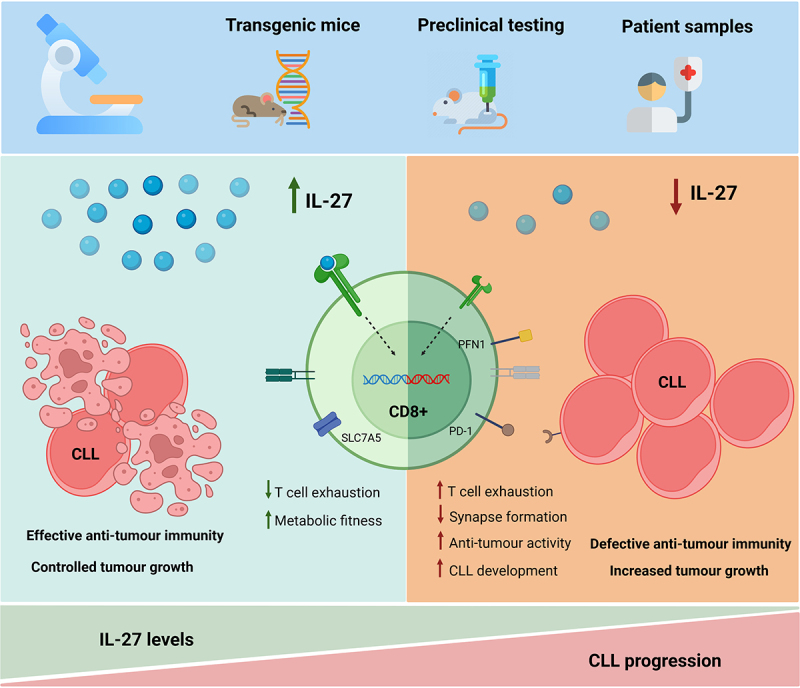
Interleukin-27 restores anti-tumor immunity in chronic lymphocytic leukemia in a T cell-mediated mechanism. Our previous publication employs murine and human samples to investigate the role of IL-27 in the development and progression of chronic lymphocytic leukemia (CLL). Among other findings, we report an inverse correlation between serum IL-27 levels and CLL development, consistent with an anti-tumor role of IL-27 in this malignancy. Pre-clinical studies corroborate this observation by showing a marked increase in tumor load in the absence of IL-27, and strongly point toward a T-cell-mediated mechanism. *in vitro* analyses uncover markers potentially involved in the aforementioned process given their key role in cytotoxicity and metabolic fitness. Finally, *ex vivo* experiments reveal that patient-derived CD8+ T cells have a significantly higher cytotoxic activity toward leukemic cells upon treatment with IL-27, highlighting the translational potential of IL-27 as a therapeutic agent to restore effective anti-tumor immunity in CLL. PD-1: programmed cell death protein 1; SLC7A5: solute carrier family 7 member 5; PFN1: profilin 1. Figure created with BioRender.com.

Overall, we demonstrated that IL-27 is an important mediator in CLL pathology (Figure 1). Moreover, we provide evidence supporting a T-cell mediated mechanism, as well as point toward and validate specific molecular targets potentially involved in the process. Further research must be performed in order to address the efficacy of this treatment in combination with other therapeutic approaches and their potential for translation into the clinic. We are hopeful that the insights revealed by this investigation will eventually contribute to the treatment quality and wellbeing of CLL patients.
